# Prognostic value of abnormally expressed lncRNAs in ovarian carcinoma: a systematic review and meta-analysis

**DOI:** 10.18632/oncotarget.14760

**Published:** 2017-01-19

**Authors:** Ping Luo, Xue-Fang Liu, Ying-Chao Wang, Nan-Di Li, Shen-Jun Liao, Ming-Xia Yu, Chun-Zi Liang, Jian-Cheng Tu

**Affiliations:** ^1^ Department of Clinical Laboratory Medicine & Center for Gene Diagnosis, Zhongnan Hospital of Wuhan University, Wuhan, China

**Keywords:** long noncoding RNAs, ovarian cancer, overall survival, prognosis, meta-analysis

## Abstract

Ovarian cancer (OC) is the most deadly gynecological cancer and it is urgently needed to find a new marker for the progress of OC. Many long noncoding RNAs (lncRNAs) have been reported to be aberrantly expressed in ovarian carcinoma, and may serve as prognostic markers. Therefore, we conducted this meta-analysis to gain a better understanding of the prognostic value of lncRNAs in patients with varian carcinoma. We systematically searched PubMed, EMBASE, and Web of Science. A total of 13 eligible studies, including 10 on clinicopathological features, 13 on prognosis were identified. Pooled hazard ratios (HRs) or odds ratios (OR) and 95% confidence intervals (95% CIs) were calculated using random- or fixed-effects models. Our results revealed that the increased expressions of 8 lncRNAs were associated with poor prognosis and the decreased expressions of 5 lncRNAs were related to poor prognosis in ovarian carcinoma. High HOTAIR expression was associated with shorter overall survival in ovarian cancer (pooled HR: 2.05, 95% CI: 1.51-2.77, *P* < 0.001). In conclusion, our meta-analysis suggested that LncRNAs could function as potential prognostic markers for ovarian cancer patients and high expression HOTAIR was associated with shorter overall survival in ovarian cancer.

## INTRODUCTION

Ovarian cancer (OC) is the most deadly gynecological cancer and caused about 140,000 women's death each year [1]. Over the past decade, the improvement of survival rates in ovarian cancer was relatively limited. The 5-year survival rate for patients diagnosed and treated at early stages (I and II) can be over 90 %, whereas, most ovarian cancer patients are diagnosed with advanced disease (stages III and IV) and the 5-year survival is less than 30 % [2]. In OC research, several prognostic biomarkers, including mRNAs, such as HuR [3], Notch3 [4] and microRNAs (miRs), such as miR-30d [5], miR-200 [6], MiR-9 and miR-223 [7] have been explored. Long non-coding RNAs (lncRNAs) have also been extensively studied because of their significant role in diagnosis, prognosis and treatment.

Long non-coding RNAs (lncRNAs) are a kind of molecules longer than 200bp that can't translate into proteins [8]. It is reported that lncRNAs is involved in various cell biological processes, such as cell proliferation, differentiation, apoptosis, and cell cycle progression [9]. More importantly, emerging evidences have revealed that lncRNAs played critical roles in tumorigenesis [10]. For example, lncRNA-ATB, a lncRNA activated by TGF- β, is significantly up-regulated in hepatocellular carcinoma and related to poor prognosis [11]. The abnormally expressed Urothelial carcinoma-associated 1 (UCA1) in HCC is correlated with TNM stage, metastasis, survival, and AFP level [12]. Highly up regulated in liver cancer (HULC) is up-regulated in gastric cancer and associated with lymph node metastasis and distant metastasis [13]. Recent studies have shown that lncRNAs also played significant roles in OC [14–16], these findings support that lncRNAs can be developed as diagnostic or prognostic biomarkers in patients with OC.

However, owing to the limitations in sample size and research programs, single study may be inaccurate and insufficient. Thus, studies should be analyzed systematically to uncover the potential clinical values of lncRNAs in OC. Until now, only reviews were conducted regarding the evaluation of the clinical values of different lncRNAs in OC, meta-analysis has yet to be performed. Therefore, with the aim to gain a better understanding of the prognostic value of lncRNAs in patients with OC, we carried out a systematic review followed by a meta-analysis of the published articles to explore the prognostic value of lncRNAs in OC.

## RESULTS

### Selection process of included studies

**Figure 1:**
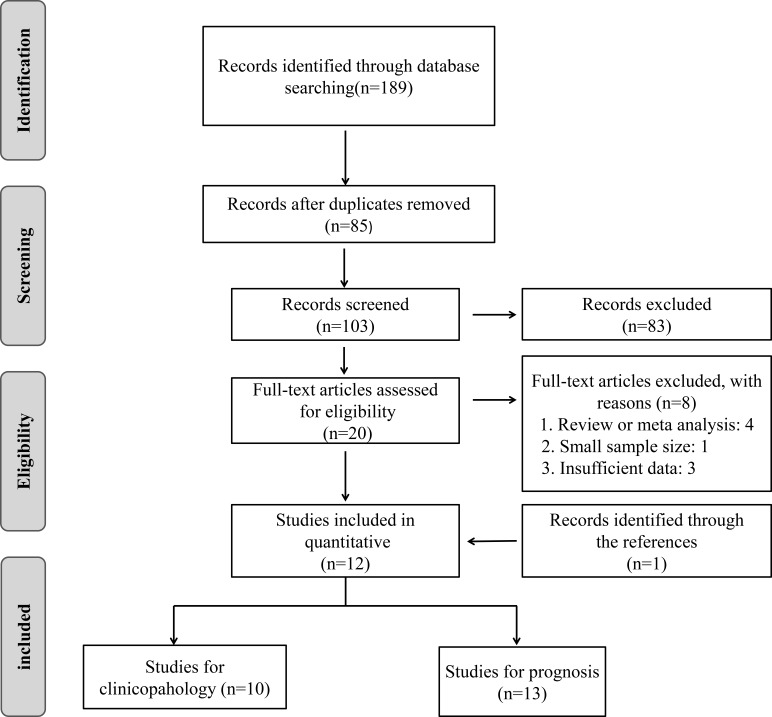
Flow diagram of study selection process

As shown in the flow diagram (Figure 1), 189 articles were retrieved from PubMed, EMBASE, and Web of Science, and 85 duplicated articles were removed, then only 103 articles were left. After we screened the titles and abstracts, 83 irrelevant articles were excluded. Subsequently, the 20 remaining full-text articles were assessed and 8 studies, including 4 review or meta-analysis, 1 with less than 60 sample numbers, 3 with insufficient data were further excluded on the basis of the exclusion criteria. Then 1 articles was identified through reviewing the references. Finally, a total of 13 articles were included in the current meta-analysis.

### Correlation of lncRNAs with clinicopathological characteristics of OC

As shown in Table 1, 9 lncRNAs were involved in the studies described clinicopathological features. HOX transcript antisense RNA (HOTAIR) [17, 18], colon cancer associated transcript 2 (CCAT2) [19], nuclear paraspeckle assembly transcript 1(NEAT1) [20], urothelial carcinoma associated 1(UCA1) [15], antisense non-coding RNA in the INK4 locus (ANRIL) [21] and TC0101441 [22] were up-regulated, whereas ASAP1-IT1, FAM215 and LINC00472 [23] were downregulated. There was no studies reported that lncRNAs were significantly associated with the age, CA125 level and ascites of patients. 3 studies [15, 18, 21] claimed that up-regulated lncRNAs were significantly related to lymph node metastasis. 6 studies showed that up-regulated lncRNAs were related to Histological grade. All studies except Teschendorf's [24]study demonstrated that lncRNAs were significantly correlated with FIGO stage.

**Table 1:** Summary of the comparison for the *p* values of the association between lncRNAs and clinicopathological features

Author, year of publication	lncRNAs	Total number	age	FIGO stage	LM	Histological grade	Residual tumor diameter	CA125	Ascites	Expression
Qiu [17] 2015	HOTAIR	68	0.318	0.006	-	0.011	0.604	0.465	0.618	Up-regulation
Qiu [18] 2014	HOTAIR	64	0.442	<0.001	<0.001	0.001	0.157	0.209	0.784	Up-regulation
Huang [19] 2016	CCAT2	109	0.702	0.002	-	0.006	-	-	-	Up-regulation
Chen [20] 2016	NEAT1	149	0.464	0.004	-	0.009	-	-	-	Up-regulation
Zhang [15] 2016	UCA1	117	0.702	0.025	0.016		-	-	-	Up-regulation
Yang [21] 2015	ANRIL	68	0.318	0.006	0.001	0.042	0.12	0.808	0.134	Up-regulation
Fu [23] 2016	ASAP1-IT1	165	0.19	-	-	0.55	-	-	-	down-regulation
Fu [23] 2016	FAM215A	165	0.028	-	-	0.004	-	-	-	down-regulation
Fu [23] 2016	LINC00472	165	0.65	-	-	0.004	-	-	-	down-regulation
Qiu [22] 2014	TC0101441	64	0.442	<0.001	-	<0.001	-	-	0.59	Up-regulation
Teschendorf [24] 2015a	HOTAIR	134	0.166	0.733	-	0.182	0.308	-	-	Up-regulation
Teschendorf [24] 2015b	HOTAIR	175	0.262	0.665	-	0.483	0.161	-	-	Up-regulation

Abbreviations: HOTAIR: HOX transcript antisense RNA; CCAT2: colon cancer associated transcript 2; NEAT1: nuclear paraspeckle assembly transcript 1; UCA1: urothelial carcinoma associated 1; ANRIL: antisense non-coding RNA in the INK4 locus

**Figure 2:**
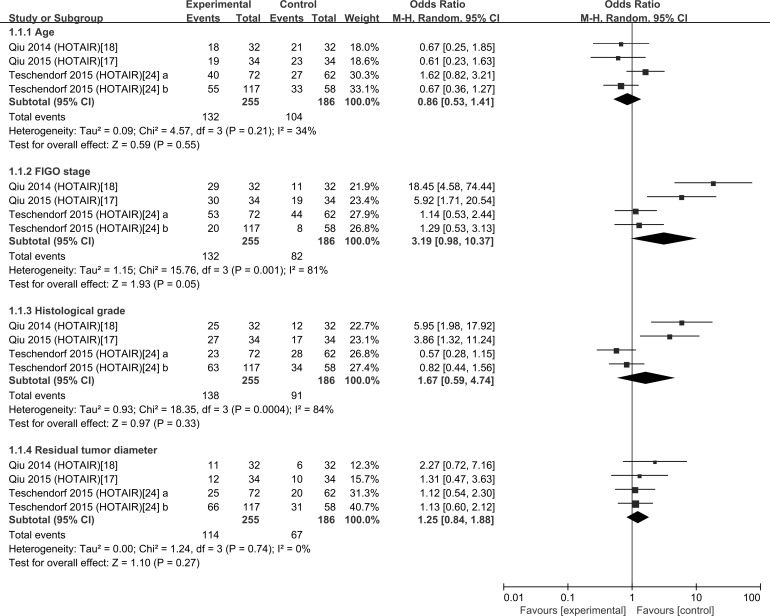
Forest plots of studies evaluating odds ratios (ORs) of HOTAIR expression and the clinicopathology of OC patients

There were four studies on HOTAIR, we then explored the relation of HOTAIR and clinicopathological features of OC patients (Figure 2). Significant heterogeneity was observed in FIGO stage (*I^2^* = 81.0%) and Histological grade *I*^2^ = 84.0%); however the heterogeneity of age (*I^2^* = 34.0%) and residual tumor diameter(*I^2^* = 0 % ) were not significant. Therefore, in this part, we compared the result between Fixed-effects model and Random-effects model (Table 2). No significant correlation between up-regulated HOTAIR and clinicopathological features in the Random-effects model was found although the *P* value of FIGO stage was 0.05 and a significant association between the expression of HOTAIR and FIGO stage was revealed in Fixed-effects model.

**Table 2:** Comparison of the result between Fixed-effects model and Random-effects model

Clinical features	Pooled OR (95% CI)		Heterogeneity	
	Random	Fixed	*I*^2^ (%)	*P*-value
Age	0.86 (0.53,1.41)	0.88 (0.60,1.29)	34	0.21
FIGO stage	3.1 (0.98,10.37)	2.35 (1.49,3.73)	81	0.001
Histological grade	1.67 (0.59,4.74)	1.22(0.83,1.79)	84	0.0004
Residual tumor diameter	1.25 (0.84,1.88)	1.26(0.84,1.88)	0	0.74

### Analysis between lncRNAs expression level and OC survival

**Figure 3:**
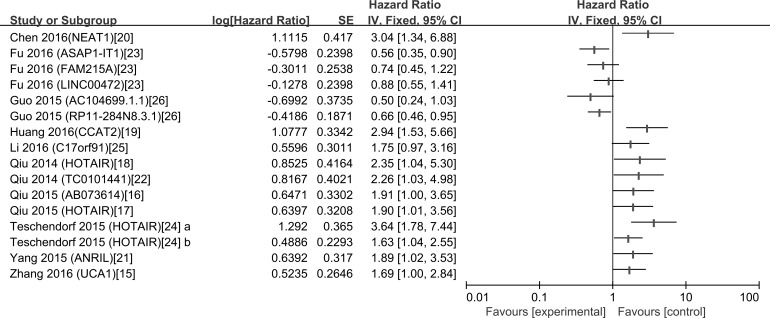
A display of Hazard ratios (HRs) of lncRNAs in OC patients The point estimate is bounded by a 95% confidence interval, and the perpendicular line represents no increased risk for the outcome. OC: ovarian cancer.

There were 13 studies explored the relationship between lncRNAs and OS of ovarian cancer patients. Among them, 10 were from China, 1 from Italy, 1 from Austria and 1 from Holland. The included studies were all retrospective and published over the recent three years. 13 different lncRNAs were associated with the prognosis of patients with OC. The characteristics of these 13 eligible studies are presented in Table 3. The increased expressions of HOTAIR [17, 18], CCAT2 [19], NEAT1 [20], C17orf91 [25], UCA1 [15], ANRIL [21], AB073614 [16] and TC0101441 [22] were associated with poor prognosis; similarly, the decreased expressions of ASAP1-IT1, FAM215, LINC00472 [23] and RP11-284N8.3.1 AC104699.1.1 [26] were related to poor prognosis (Figure 3). With all the lncRNAs, HOTAIR generated the highest hazard ratio (HR) of 3.64 [24]; by contrast, AC104699.1.1 [26] exhibited the lowest HR of 0.50.

**Table 3:** Summary of lncRNAs used as prognostic biomarkers of ovarian cancer

Author, year of publication	Country	lncRNAs	Total number (High/low)	Cutoff	Method	Internal reference	Outcome	Follow-up (month)	Quality score
Qiu [17] 2015	China	HOTAIR	34/34	Median	qRT-PCR	GAPDH	OS	100	8
Qiu [18] 2014	China	HOTAIR	32/32	Median	qRT-PCR	GAPDH	OS	80	8
Huang [19] 2016	China	CCAT2	55/54	Median	qRT-PCR	GAPDH	OS	60	7
Li [25] 2016	China	C17orf91	−/−	-	qRT-PCR	GAPDH	OS/PFS	80	7
Chen [20] 2016	China	NEAT1	74/75	Median	qRT-PCR	GAPDH	OS	70	8
Zhang [15] 2016	China	UCA1	59/58	Median	qRT-PCR	RUN6	OS	80	8
Yang [21] 2015	China	ANRIL	34/34	Median	qRT-PCR	GAPDH	OS	100	8
Qiu [16] 2015	China	AB073614	38/37	-	qRT-PCR	GAPDH	OS	60	7
Fu [23] 2016	Italy	ASAP1-IT1	81/84	Median	qRT-PCR	GAPDH	OS/PFS	144	8
Fu [23] 2016	Italy	FAM215A	84/77	Median	qRT-PCR	GAPDH	OS/PFS	144	8
Fu [23] 2016	Italy	LINC00472	82/84	Median	qRT-PCR	GAPDH	OS/PFS	144	8
Qiu [22] 2014	China	TC0101441	32/32	Median	qRT-PCR	GAPDH	OS	80	8
Teschendorf [24]2015a	Austria	HOTAIR	72/62	-	qRT-PCR	TBP	OS	60	7
Teschendorf [24] 2015b	Holland	HOTAIR	117/58	-	qRT-PCR	TBP	OS	60	7
Guo [26] 2015	China	RP11-284N8.3.1	199/200	-	DEGseq	-	OS/PFS	160	7
Guo [26] 2015	China	AC104699.1.1	199/200	-	DEGseq	-	OS/PFS	160	8

Abbreviations: OC: ovarian cancer; qRT-PCR: quantities reverse transcription polymerase chain reaction; GAPDH: glyceraldehyde 3-phosphate dehydrogenase; TBP: TATA-box bingding protein; OS: overall survival; PFS: prognostic free survival

**Figure 4:**
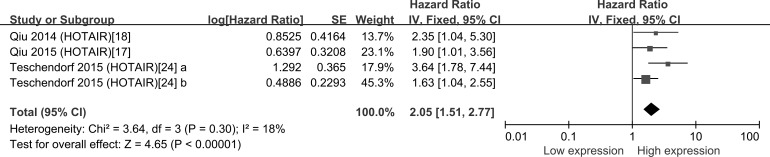
Forest plots for the association between HOTAIR expression and OS of OC patients The point estimate is bounded by a 95% confidence interval, and the perpendicular line represents no increased risk for the outcome. OS: overall survival; OC: ovarian cancer.

Four studies investigated the association between the expression of HOTAIR and OS in a total number of 441 patients, we then conducted a meta-analysis on the relationship of HOTAIR expression and the overall survival (OS) of patients with ovarian cancer; As the heterogeneity was not significant (*I^2^* = 18.0%, *P* = 0.30), a fixed effects model was applied. The model revealed that high HOTAIR expression was associated with shorter OS in ovarian cancer (pooled HR: 2.05, 95% CI: 1.51-2.77, *P* < 0.001) (Figure 4).

### Sensitivity analysis and publication bias

**Figure 5:**
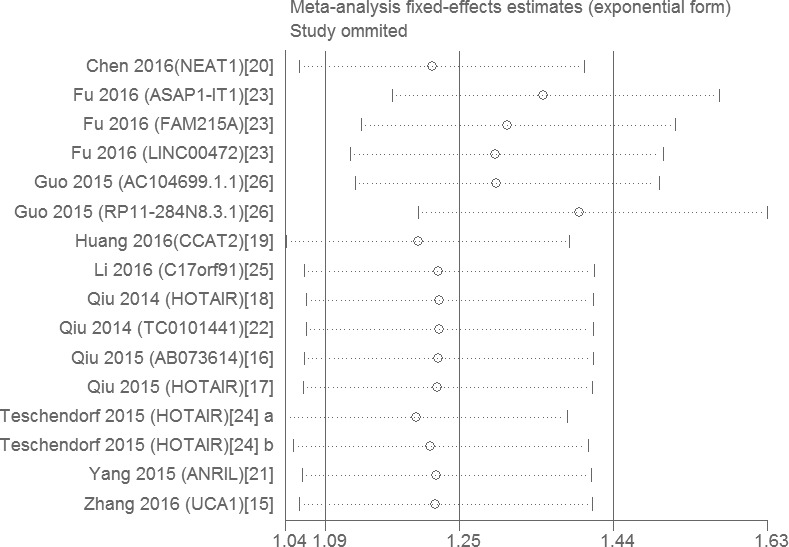
Sensitivity analysis of the influence of each individual study on the pooled HRs by omitting individual studies

Stata11.0 software was used to perform sensitivity analysis to assess whether the individual studies affected the overall results. The results revealed that individual study had little influence on our final results (Figure 5) and validated the reliability of our results. Egger's test was used to evaluate the publication bias. The *P* value of the Egger's test was 0.015, which was > 0.01 but < 0.05, it indicated that there was publication bias existed in this meta-analysis.

## DISCUSSION

With the development of technology, increasing evidence demonstrated that aberrant expression of lncRNAs were associated with clinical outcomes for cancer patients. Multiple studies have revealed that lncRNAs were involved in the onset and progression of cancer including apoptosis, metastasis, migration, and other clinical outcome [27]. LncRNAs may exert their roles through the following ways: (1) interfering in the expression of the adjacent encoding protein gen [28]; (2) binding with functional protein [29]; (3) acting as the precursors of miRNAs and affecting on target genes of miRNA [30]; (4) regulating signaling pathway through combing with chromosome [31].

Recently, more and more studies found that aberrant expression of multiple lncRNAs were involved in the tumorigenesis and may have prognostic value for OC [32]. Gao *et al*. [33] demonstrated that HOST2 was overexpressed in ovarian cancer and the tumorigenic effects of HOST2 were dependent on its ability to act as a molecular sponge for let-7b. Zou *et al*. [34] found that MALAT1 was up-regulated in ovarian cancer tissue and promoted SK-OV-3 cell proliferation and invasion. In order to have a better understanding between lncRNAs and OC, further to identify a prognostic marker for OC, we carried out this comprehensive systematic review and meta-analysis of the current literature.

Up to now, two meta-analyses [35, 36] evaluated the association between lncRNAs and cancer survival involved OC as one of the cancer sites. Both of these two studies focused on exploring association between lncRNA UCA1 or MALAT-1 and OS in all human cancers, ovarian cancer is just one of them. Our article, on the other hand, with quite different goal, was mainly focused on all lncRNAs that related to OC. Therefore the present meta-analysis is the first to explore the association between lncRNAs expression and clinical features of OC comprehensively.

In the present meta-analysis, we found that the increased expressions of 8 lncRNAs were associated with poor prognosis; similarly, the decreased expressions of 5 lncRNAs were related to poor prognosis in OC. Summary of Hazard ratios (HRs) of lncRNAs in OC patients showed that HOTAIR generated the highest hazard ratio (HR) of 3.64 [24]; by contrast, AC104699.1.1 [26] exhibited the lowest HR of 0.50. HOTAIR was the most investigated lncRNA and reported by four studies, the pooled HR showed that high HOTAIR expression was significantly associated with shorter OS in ovarian cancer. We then explored the relation between HOTAIR expression and clinicopathological characteristics. Because of the different *I*^2^ value among the subgroup, Fixed-effects model and Random-effects model were both used. We found that high HOTAIR expression was discovered to be associated with high FIGO stage in Fixed-effects model, in the Random-effects model, although the result was not significant, the *P* value was 0.05, which is almost smaller than 0.05, representing a high likelihood of a repeatable difference.

Our meta-analysis demonstrated that HOTAIR was correlated with OC prognosis but not Histological grade. Qiu *et al*. [17, 18] reported that high HOTAIR expression was associated with high histological grade (G3). However, Teschendorf *et al*. [24] did not find any association between increased HOTAIR expression and grade, the first two studies [17, 18] included ovarian carcinoma patients with serous or epithelial subtype, whereas the third study [24] included all subtypes (serous, mucinous, endometrioid, clear cell) as well as fallopian tube and non-classifiable tumors, which may explain this discrepancy. HOTAIR is an lncRNA expressed from the *HOXC* locus on chromosome 12 [37]. Studies reported that HOTAIR acted as a scaffold lncRNA binding to both PRC2 and LSD1 histone modification complexes through its 5′ and 3′ domains respectively [38].

HOTAIR is aberrantly expressed in a variety of human cancers, such as breast cancer [39], colorectal cancer [40], laryngeal squamous cell carcinoma [41], and liver cancer [42]. The up-regulation of the HOTAIR expression was related to poor prognostic outcome of different cancers, those findings in consist with our results. HOTAIR was the most investigated lncRNA in OC, therefore, articles explored HOTAIR in OC is possibly the most promising.

It should be stressed that there were limitations in our analysis. Firstly, the cutoff value and method for low or high levels of lncRNA varied in different studies, which may cause the heterogeneity of results; secondly, different lncRNAs were used to assess the prognosis of OC, leading to lack of specific OC-related lncRNA for clinical evaluation; thirdly, since in most cases there was only one study for each lncRNA, we may overestimated the prognostic value of each lncRNA; fourthly, publication bias is a concern, language restriction of our analysis to published studies written in English may result in publication bias, furthermore, the fact that negative results are more difficult to be published maybe also contributed to the publication bias.

In conclusion, our study for the first time evaluated the correlation between lncRNAs and clinical characteristics of patients with OC. Despite the existence of limitations, the present study revealed that lncRNAs could be considered as biomarkers for FIGO stage and Histological grade. We also found that lncRNAs could be used as potential prognostic markers for OC and high HOTAIR expression could predict poor survival among patients with OC. However, in view of the limitation of individual studies about lncRNAs, in future, further comprehensive, large-scale, and good quality studies should be conducted to confirm our findings and thus promote the clinical utility of lncRNAs in evaluating ovarian patients’ prognosis

## MATERIALS AND METHODS

### Search strategy

We searched the databases PubMed, EMBASE, and Web of Science for studies that relevant about the prognostic value of lncRNA in OC. The search ended in November 2016. The search terms used were:(“Long noncoding RNA”, “lncRNA”, “LincRNA”, “Long ncRNA”, “Long intergenic non-coding RNA”) AND (“ovarain cancer”, “ovarain tumor”, “ovarain carcinoma”, “ovarain neoplasm”). Additionally, we screened the references of retrieved relevant articles to identify potentially eligible literatures.

### Inclusion and exclusion criteria

The inclusion criteria were as follows: (1) studies investigated the expression of lncRNAs in OC, (2) studies described the relationship between lncRNA expression and overall survival (OS) and sufficient data to estimate hazard ratios (HRs) for survival rates and their 95% confidence interval, (3) studies published in English. Exclusion criteria were as follows: (1) studies without usable data, (2) duplicate publications, (3) sample cases fewer than 60, (4) reviews, letters, single case reports, (5) animal studies, (6) HRs calculated on the basis of multiple lncRNAs.

### Data extraction and quality assessment

Two investigators (Ping Luo and Xue-Fang Liu) retrieved the data independently and the following information was extracted: (1) publication information: first author, year of publication; (2) patients’ characteristic information: study population, sample size, and follow-up duration; (3) lncRNA information: detection methods, cut-off definition, and relationship between lncRNAs and survival outcome or clinicopathological features; (4) HRs, 95 % CI for survival analysis. Studies explored the expression level of lncRNAs in different data set were considered to be different studies. The quality assessment of non-randomized studies is an important component of a thorough meta-analysis of non-randomized studies, so Newcastle-Ottawa Scale (NOS) criteria was used to assess the quality of the included studies [43]. Ping Luo and Xue-Fang Liu independently assessed the Quality of the included studies. Any disagreements were resolved through discussing with Ying-Chao Wang.

### Statistical analysis

All analyses were performed using the STATA software version 11.0 (Stata Corporation, College Station, Texas, USA) or Review Manager version 5.3. HR and 95% CIs were used to assess the association between lncRNAs and OS in OC, as to the relation between lncRNAs and clinical features, ORs and 95% CIs were used. We got HRs with their 95% CIs directly from data in articles or from Kaplan-Meier survival curves using Engauge Digitizer version 4.1 [44]. Fixed-effects model was used when there was no significant heterogeneity (I^2^≤50% or *P*≥0.05) between studies, otherwise, the random-effects model was used [45]. An observed HR > 1 implied a worse survival for the group with elevated lncRNA expression and an observed HR < 1 implied a better survival for the group with increased lncRNA expression. Sensitivity analysis was conducted by omitting the study sequentially. Publication bias was evaluated using the funnel plot with Egger's test, *P* < 0.05 was considered statistically significant.
